# Retinal Cell Death Caused by Sodium Iodate Involves Multiple Caspase-Dependent and Caspase-Independent Cell-Death Pathways

**DOI:** 10.3390/ijms160715086

**Published:** 2015-07-03

**Authors:** Jasmin Balmer, Rahel Zulliger, Stefano Roberti, Volker Enzmann

**Affiliations:** 1 Department of Ophthalmology, Inselspital, University of Bern, Bern 3010, Switzerland; E-Mails: jasmin.balmer@ndcn.ox.ac.uk (J.B.); s.roberti@gmx.net (S.R.); 2 Nuffield Laboratory of Ophthalmology, Nuffield Department of Clinical Neurosciences, University of Oxford, Oxford OX3 9DU, UK; 3 Department of Cell Biology, the University of Oklahoma Health Sciences Center, University of Oklahoma, Oklahoma City, OK 73104, USA; E-Mail: rahel-zulliger@ouhsc.edu; 4 Department for Clinical Research, University of Bern, Bern 3010, Switzerland

**Keywords:** sodium iodate, cell death, retinal pigment epithelium, photoreceptors, apoptosis, necrosis, *in vivo*, *in vitro*

## Abstract

Herein, we have investigated retinal cell-death pathways in response to the retina toxin sodium iodate (NaIO_3_) both *in vivo* and *in vitro.* C57/BL6 mice were treated with a single intravenous injection of NaIO_3_ (35 mg/kg). Morphological changes in the retina post NaIO_3_ injection in comparison to untreated controls were assessed using electron microscopy. Cell death was determined by TdT-mediated dUTP-biotin nick end labeling (TUNEL) staining. The activation of caspases and calpain was measured using immunohistochemistry. Additionally, cytotoxicity and apoptosis in retinal pigment epithelial (RPE) cells, primary retinal cells, and the cone photoreceptor (PRC) cell line 661W were assessed *in vitro* after NaIO_3_ treatment using the ApoToxGlo™ assay. The 7-AAD/Annexin-V staining was performed and necrostatin (Nec-1) was administered to the NaIO_3_-treated cells to confirm the results. *In vivo*, degenerating RPE cells displayed a rounded shape and retracted microvilli, whereas PRCs featured apoptotic nuclei. Caspase and calpain activity was significantly upregulated in retinal sections and protein samples from NaIO_3_-treated animals. *In vitro*, NaIO_3_ induced necrosis in RPE cells and apoptosis in PRCs. Furthermore, Nec-1 significantly decreased NaIO_3_-induced RPE cell death, but had no rescue effect on treated PRCs. In summary, several different cell-death pathways are activated in retinal cells as a result of NaIO_3_.

## 1. Introduction

Age-related macular degeneration (AMD) is the main cause of blindness in the elderly in industrialized countries. The dry form of AMD is characterized by the disease-associated formation of drusen, which represent debris accumulating between the Bruch’s membrane and the basal lamina of the retinal pigment epithelium (RPE). The resulting geographic atrophy of the RPE layer is followed by vision loss due to photoreceptor degeneration in the overlying region of the sensory retina, the so called macula. Currently, no treatment is available to regain lost vision in patients suffering from dry AMD. The disease mechanisms are poorly understood, thus aggravating the search for potential therapeutic approaches.

The sodium iodate (NaIO_3_) model of pharmacologically induced retinal degeneration, known to display AMD-associated features, was widely used to further understand the cell-death mechanisms in RPE and photoreceptors (PRC) [1,2,3]. In this model, the progression and onset of the degeneration can be modulated, facilitating the design of a potential therapeutic strategy to repopulate the damaged RPE layer [4,5]. NaIO_3_ is an oxidizing compound that has been shown to be specifically toxic for RPE cells [6]. Under physiological conditions, RPE cells exhibit various crucial retinal functions and the undisturbed unit of RPE and PRCs provides the basis for a normal visual perception (see review [7]). Therefore, without functional underlying RPE cells, the photoreceptors are unable to survive resulting in vision loss [8].

The NaIO_3_ mouse model has been well characterized with respect to the loss of RPE cells and the death of PRCs, resulting in a thinning of the outer nuclear layer (ONL) and a reduction in visual functions [9]. To date, it is controversially discussed whether NaIO_3_ cytotoxicity acts exclusively on RPE cells with PRCs dying secondarily as a consequence of the loss of functional RPE or whether it directly affects both retinal cell types. Previous reports have supported a direct effect on RPE cells, in which the basal plasma membrane is destroyed following NaIO_3_ administration, resulting in damage to intracellular organelles [10]. Additionally, it has been suggested that NaIO_3_ can cross-react with melanin, increasing the turnover of glyoxylate, which is cytotoxic to RPE cells [11]. NaIO_3_ is also known to have an inhibitory action on the activity of crucial enzymes (triose phosphate dehydrogenases, lactate dehydrogenase) [12]. Other reports described an altered adhesion between the neurosensory retina and the RPE layer in response to NaIO_3_ intoxication [13,14]. Recent studies have indicated a direct effect of NaIO_3_ on the sensory retina [15,16]. NaIO_3_ was thereby demonstrated to alter gene expression *in vivo* by down-regulation of RPE cell-specific markers as early as 24 h post-injection, concomitantly with an increase in the expression of the pro-apoptotic *Bax* gene within the neurosensory retina. Furthermore, it was demonstrated that NaIO_3_ treatment *in vitro* increased levels of reactive oxygen species (ROS) in the 661W cone photoreceptor cell line [17,18]. However, no report to date has defined whether caspase-dependent or caspase-independent cell-death pathways are involved in NaIO_3_-induced RPE and PRC death *in vivo*; this knowledge is crucial for the design of a experimental strategy to intervene with NaIO_3_-induced photoreceptor cell loss.

Apoptosis is the conventional form of cell death, in which caspases are activated, resulting in nuclear fragmentation and chromosome condensation. To date, growing evidence exists supporting the contribution of nonconventional (caspase-independent) cell-death mechanisms to the progression of neurodegenerative diseases [19,20]. Activation of calpains (calcium-dependent proteases) and cathepsins (lysosomal proteases) in fact has been reported in degenerative processes [21,22]. Calpains are ubiquitously expressed and highly activated under stress conditions in response to an increased influx of ions through cGMP-gated cation channels. They have been reported to be involved in PRC cell death in the *rd1* mouse model, or retinitis pigmentosa [23], as well as in P23H and S334ter rhodopsin mutant rats [24]. The underlying mechanism can be either caspase-dependent or caspase-independent. Necrotic cell death (necrosis), on the other hand, is a less defined and uncontrolled death mechanism that does not involve the activation of conventional cell death key players.

In the presented study, with the aim to characterize the NaIO_3_ model that displays AMD-associated features, we assessed retinal changes following the administration of NaIO_3_ *in vivo* and *in vitro*. Thereby, it could be shown that, in PRCs of NaIO_3_-injected animals, caspases as well as calpain proteases were upregulated on the protein level. Furthermore, we provide evidence that, in RPE cells, NaIO_3_ treatment resulted in necrosis in the absence of caspase-dependent conventional cell death, whereas it triggered caspase-dependent apoptosis in the cone photoreceptor cell line 661W. Consistently, incubation of NaIO_3_-treated RPE cells with necrostatin 1 (Nec-1), an inhibitor of nonconventional necrosis, was cytoprotective, but the compound did not prevent NaIO_3_-induced PRC cell death. In summary, NaIO_3_ triggered both RPE and PRC degeneration via different pathways involved in cell death induction and execution. Therefore, a combinatory treatment might be the most straightforward approach to modulate this complex model of retinal degeneration, opening new possibilities to use the NaIO_3_ mouse model to design a therapeutic strategy to prevent vision loss.

## 2. Results

### 2.1. Ultrastructural Alterations in the Retina Following NaIO_3_ Administration

**Figure 1:**
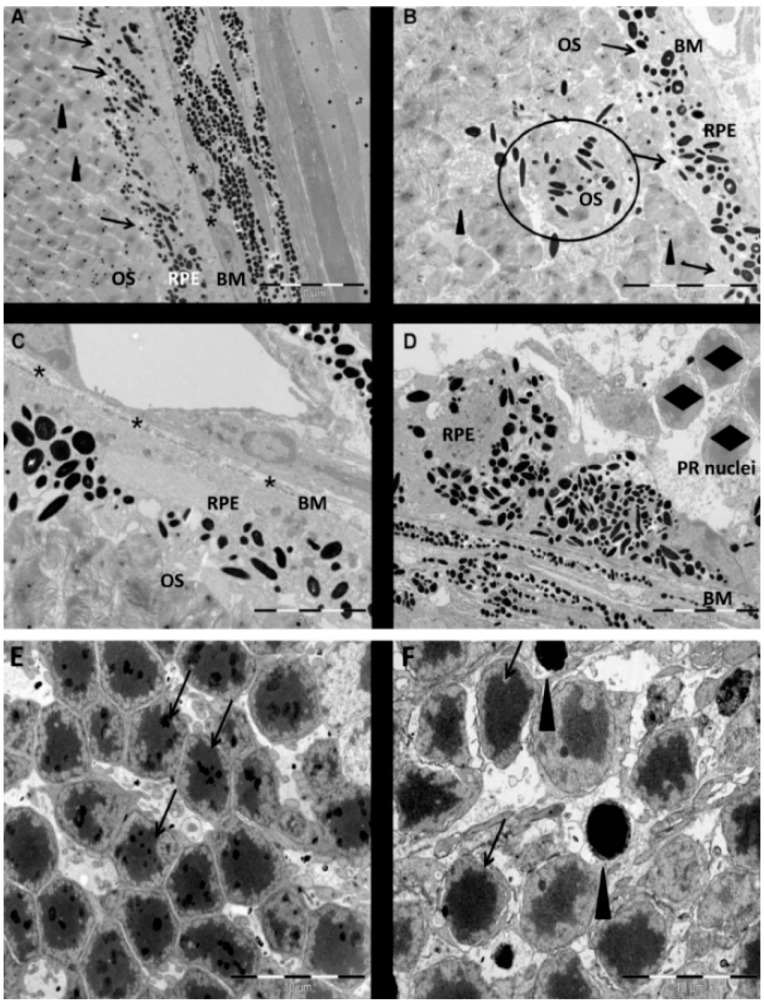
Time-dependent alterations in the outer retinal structure of mice induced by the retina toxin sodium iodate (NaIO_3_). (**A**) Under physiological conditions, retinal pigment epithelial (RPE) cells display apically located melanin granules and microvilli (arrows) engulfing photoreceptor outer segments (arrowheads). Normal Bruch’s membrane is marked by asterisks; (**B**) Three days post-injection of 35 mg/kg NaIO_3_, microvilli were not present on the RPE surface (arrows) and RPE cells as well as melanin granules (black circle) are displaced into the layer of the outer segments (arrowheads); (**C**) The RPE monolayer was disturbed and the Bruch’s membrane appeared swollen (asterisks); (**D**) The epithelial monolayer was completely disrupted and individual RPE cells showed a rounded phenotype two weeks after injection. The remaining photoreceptors (PRC) nuclei (diamonds) were located close to the RPE cells, as the outer segments were absent; (**E**) In the controls, PRC nuclei were evenly distributed and displayed a dark center and a bright rim (arrows); (**F**) Nuclei of PRC in NaIO_3_-treated mice revealed nuclear condensation (arrowheads), but normal nuclei (arrows) as well as organelle swelling and discontinuities in nuclear and plasma membrane (asterisk) were also seen at day 3 post-injection. Scale bar: 10 µm (**A**,**B**,**E**,**F**), 5 µm (**C**,**D**).

Systemically administered NaIO_3_ (35 mg/kg) resulted in a rapid destruction of RPE cells at the ultrastructural level (Figure 1). Healthy RPE cells displayed a defined polarity and were characterized by the presence of microvilli on the apical surface (arrow) and a normal Bruch’s membrane (asterisks). The microvilli are wrapped around the outer segments of the photoreceptors (arrowhead; Figure 1A), corresponding to the physiological situation in which shed outer segments are phagocytized by RPE cells. Three days post-injection (PI) of NaIO_3_, microvilli were visibly retracted (arrows; Figure 1B). The melanin pigment granules were not arranged apically within the cell, as they were under physiological conditions, but were observed to be distributed in a non-polarized manner. PRC outer segments were not engulfed by microvilli (arrowheads), and individual RPE cells were detached and lost their cellular integrity (Figure 1B). Furthermore, RPE cells were occasionally found displaced into the photoreceptor layer (black circle). The Bruch’s membrane (asterisks) and choriocapillaris appeared swollen, but were still intact (Figure 1C). Two weeks post induction of cell death, the remaining RPE cells displayed a rounded cell shape, and the RPE monolayer was disrupted and directly located adjacent to the PRC nuclei (diamonds, Figure 1D). PRCs were also affected in response to NaIO_3_. The nuclei of healthy PRCs in the control were characterized by a bright rim and a dark center (arrows, Figure 1E), whereas nuclei of PRCs of NaIO_3_-treated mice at day 3 PI revealed nuclear condensation, which is a sign of apoptosis (arrowheads, Figure 1F). However, organelle swelling and discontinuities in nuclear and plasma membranes (asterisks, Figure 1F) were also observed at day 3 PI, indicating a contribution of necrotic cell death to NaIO_3_-induced PRC death [25]. This was in contrast to the control samples, which never contained apoptotic, but only individual necrotic PRC nuclei. Additionally, treatment with NaIO_3_ damaged basal infoldings which appeared to be fewer and dilated. This was also different from the control sample with its regular basal infoldings. Similar but more pronounced damage has also been reported in response to the administration of higher concentrations of NaIO_3_ (70 mg/kg) that—compared to our dose—resulted in a complete loss of RPE cells and consequently in a free Bruch’s membrane [26].

### 2.2. Calpain and Caspases Are Involved in NaIO_3_-Induced Photoreceptor Cell Death in Vivo

**Figure 2:**
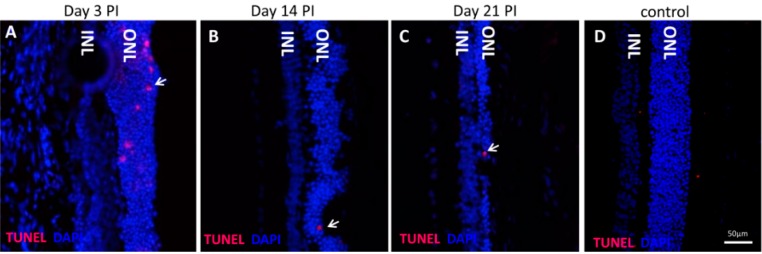
NaIO_3_ induces cell death in PRCs in a time-dependent manner. (**A**) TUNEL-positive PRCs (red) were detected at day 3 post-injection; (**B**) The number of TUNEL-positive cells decreased until day 14 post-injection, indicating that the peak of apoptosis was past; (**C**) Two-to-three rows of PRC nuclei remained three weeks post-induction of the degeneration; (**D**) No TUNEL-positive cells were found in the ONL of the control samples. ONL = Outer nuclear layer; INL = Inner nuclear layer. Arrows mark individual TUNEL-positive cells.

TUNEL-positive cells were absent in the control sections (Figure 2D), but were detectable after NaIO_3_ treatment starting from day 3 PI (Figure 2A). TUNEL-positive cells (red) were restricted to the outer nuclear layer (ONL), in which photoreceptor nuclei are located, indicating a cell-specific effect (primary or secondary) of NaIO_3_ administration (Figure 2). TUNEL positivity decreased over time (Figures 2B,C). Quantification revealed that only 0.5% ± 0.2% of all photoreceptors were TUNEL positive at day 3 PI, indicating that photoreceptor degeneration is rather slow to progress after NaIO_3_ treatment (35 mg/kg).

**Figure 3:**
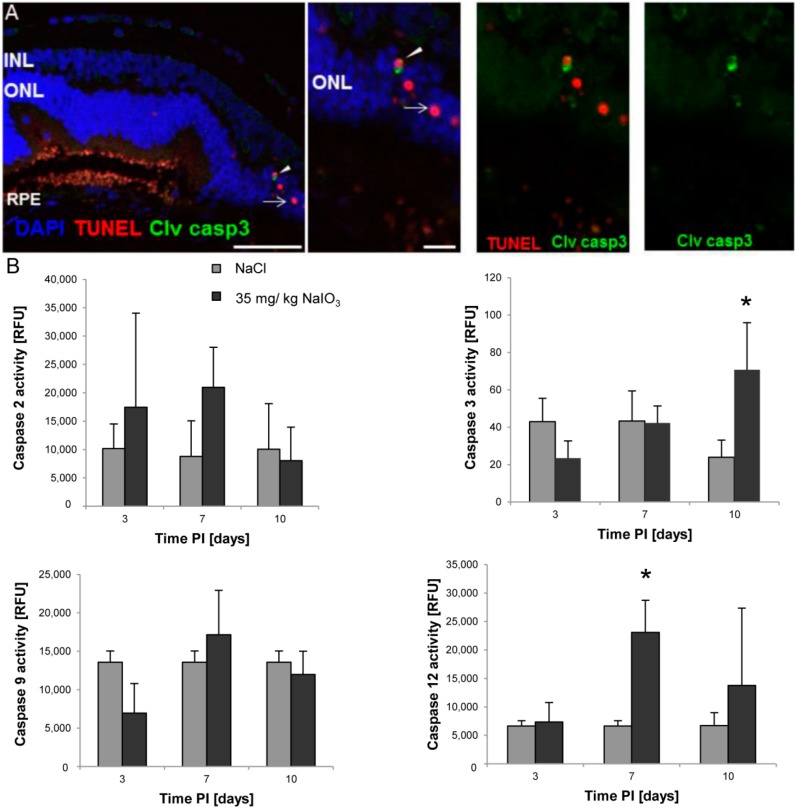
Caspase-dependent cell-death mechanisms are involved in PRC death in response to NaIO_3_. (**A**) Few cleaved caspase-3-positive cells (green) could be visualized in the ONL at day 3 post injection. A low number of cells shows co-localization with TUNEL positivity in red (arrowheads), whereas other caspase-3-positive cells were not TUNEL-positive (arrow), representing an early stage of cell death. Scale bar = 50 µm in the overview image, 10 μm in the magnification images. GCL = Ganglion cell layer;INL = Inner nuclear layer; ONL = Outer nuclear layer; RPE = Retinal pigment epithelium; (**B**) In protein samples of retinas of NaIO_3_-treated mice, a significant upregulation (*) of caspase-3 (day 10) and caspase-12 (day 7) was detectable compared to the expression level in the untreated control lysates.

As TUNEL staining does not exclusively detect apoptosis, but also other forms of cell death characterized by DNA fragmentation, retinal sections of NaIO_3_-treated animals were assessed for the activation of the key executioner caspase of conventional cell death, cleaved caspase-3 (Figure 3). No expression was detectable in the control samples (not shown), but few cleaved caspase-3-positive cells (green cells in Figure 3A) were detected in the ONL of NaIO_3_-treated mice at day 3. The low number is consistent with the low TUNEL positivity observed in retinal sections. In fact, all caspase-3-positive cells showed co-localization with TUNEL staining (arrowheads; Figure 3A), whereas not all TUNEL-positive cells were also positive for activated caspase-3 (arrows, Figure 3A,B). The result indicates that caspase activity represents the early stages of PRC cell death. Furthermore, retinal protein lysates of NaIO_3_ and NaCl-injected animals were assessed for the activation of several caspases (Figure 3B). Caspase-2 and -9 were not significantly upregulated at any of the observed time points. Executioner caspase-3, however, was significantly upregulated compared to the controls at day 10 PI (3.0-fold; *p* = 0.001), as was caspase-12, the protease that mediates endoplasmic reticulum (ER)-specific cell death [27] at day 7 PI (3.4-fold; *p* = 0.002). The measured increase in activity indicates the involvement of the canonical cell-death pathway, but does not exclude additional contributions of caspase-independent cell-death mechanisms.

**Figure 4:**
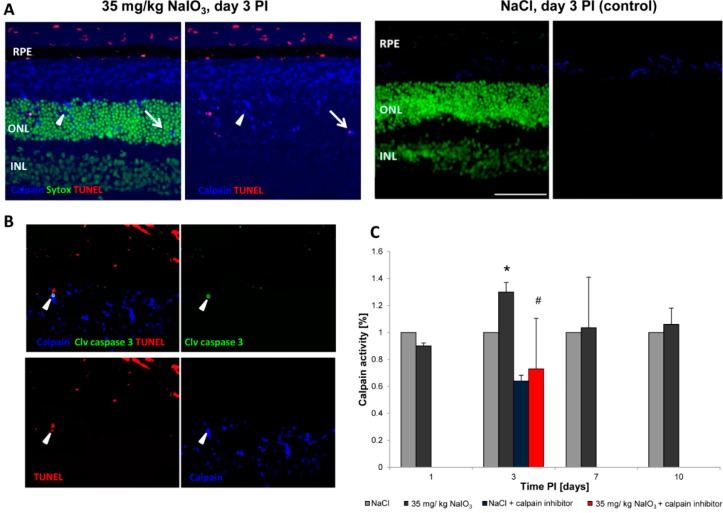
Caspase-independent cell-death mechanisms are also involved in PRC death in response to NaIO_3_. (**A**) Calpain is activated in degenerating PRCs. At day 3, calpain activity (blue, arrowhead) was detected exclusively in the ONL (**left panel**). No activity was detectable in the control sections (**right panel**). Individual calpain-expressing cells were also TUNEL-positive (arrow), representing a late stage cell death; (**B**) Individual TUNEL-positive PRCs (red) that expressed activated calpain (blue) were also caspase-3 (green) positive (arrowhead). Scale bar = 50 μm; (**C**) A significant upregulation of calpain activity was measured in retinal lysates of NaIO_3_-treated animals at day 3 post-injection (* *p* < 0.05). This upregulation could be abolished if lysates were incubated with a calpain inhibitor prior to exposure to the substrate (# *p* < 0.05).

To investigate the involvement of nonconventional cell-death pathways, we assessed the retinal samples of NaIO_3_-treated animals for the presence of activated calpains (Figure 4), proteases known to induce neurodegenerative processes. In retinal sections of NaCl-injected control animals, no positive staining for activated calpain was observed in the ONL (Figure 4A, right panel). However, in NaIO_3_-injected mice, numerous PRCs were positive for activated calpain, which is characterized by a blue staining localized at nucleus and cytoplasm (Figure 4A, arrowhead). The highest percentage of calpain positivity in the ONL (24.1% ± 1.7% of all PRCs) was observed at day 3 PI. Few calpain-positive cells (5.7% ± 4%) were also TUNEL-positive (Figure 4A, arrow), indicating that cells in which calpain was activated will undergo cell death. Furthermore, the activation of calpain was confirmed at the protein level (Figure 4C). In retinal lysates of treated animals, calpain activity was upregulated significantly (1.3-fold) in comparison to the controls at day 3 PI (*p* = 0.05). The increase was abolished (0.73-fold of wild type enzyme activity; *p* = 0.02) when the samples were incubated with the calpain inhibitor Z-LLY-FMK before adding the calpain substrate. In order to determine whether calpain and caspase-3 were activated in the same cells, co-staining was performed. Individual calpain-positive cells were also positive for cleaved caspase-3 (Figure 4B, arrowhead), indicating a concomitant execution of caspase-dependent and caspase-independent mechanisms after NaIO_3_ treatment or a caspase-dependent mode of action of calpain.

### 2.3. NaIO_3_ Induces Necrosis in RPE Cells and Apoptotic Cell Death in 661W Cells in Vitro

**Figure 5:**
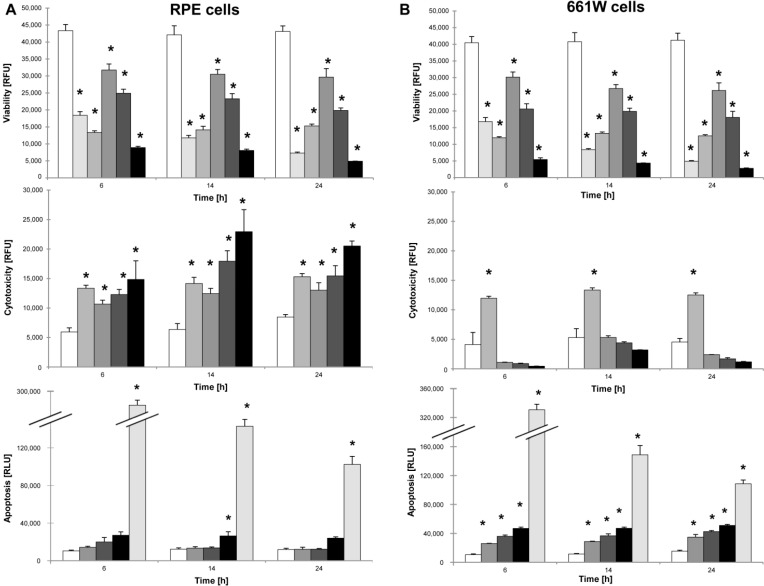
NaIO_3_ is cytotoxic for RPE cells, but induces apoptosis in PRC *in vitro*. (**A**) NaIO_3_ triggers a time- and dose-dependent loss in RPE cell viability (**upper panel**). This was mainly induced by cytotoxicity, as measured at all concentrations and at all of the time-points (**center panel**). No significant increase in apoptosis was found compared to the untreated control samples (**lower panel**); (**B**) NaIO_3_ induces a time- and dose-dependent loss of 661W cell viability (**upper panel**). No significant cytotoxicity after NaIO_3_ treatment was detectable for these cells (**center panel**). Apoptosis was significantly increased compared to the untreated controls at all concentrations (**lower panel**). Staurosporine and sonication were used as positive controls for apoptosis and necrosis, respectively. (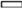 cells, 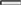 + 1 μM staurosporine, 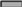 + 100% sonication, 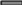 + 6 mM NaIO_3_, 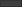 + 12 mM NaIO_3_, 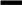 + 48 mM NaIO_3_); * *p* < 0.05.

Cell viability was measured to investigate the direct effect of NaIO_3_ on primary RPE cells, immortalized PRCs (cone photoreceptor-derived 661W cells) as well as on freshly digested neurosensory retina *in vitro*. Cells were incubated with different concentrations of NaIO_3_ and the viability was assessed at 6, 14, and 24 h post-exposure. A significant dose-dependent loss of cell viability (*p* ≤ 0.01) was confirmed for all cell types at any time (Figure 5A,B upper panels; Figure S1). For control purposes, caspase-dependent apoptosis was induced by staurosporine, and necrotic-like plasma membrane rupture was stimulated by sonication.

To assess differences in the induced cell-death mechanisms, necrotic (cytotoxic) and apoptotic (caspase-dependent) cell death was assessed in NaIO_3_-treated RPE and 661W cells. A significant increase in necrotic cell death was observed in RPE cells (*p* ≤ 0.05) at each time point after exposure to NaIO_3_ (Figure 5A, center panel). In contrast, apoptosis was absent following the treatment of RPE cells (Figure 5A, lower panel), with the exception of the highest NaIO_3_ concentration (48 mM) at 14 h after exposure (*p* = 0.01). In 661W cells on the other hand, no significant cytotoxicity was detectable after treating 661W cells with NaIO_3_ compared to the induced cytotoxicity by sonication (Figure 5B, center panel). This indicates that cone photoreceptors do not die by primary necrosis. A significant increase in apoptotic cell death (*p* ≤ 0.01) was, however, observed in 661W cells compared with the untreated controls (Figure 5B, lower panel) at all concentrations and all time points post-exposure. In conclusion, RPE cells underwent necrotic cell death in response to NaIO_3_, whereas caspase-dependent apoptosis was triggered in the 661W cone photoreceptors.

**Figure 6:**
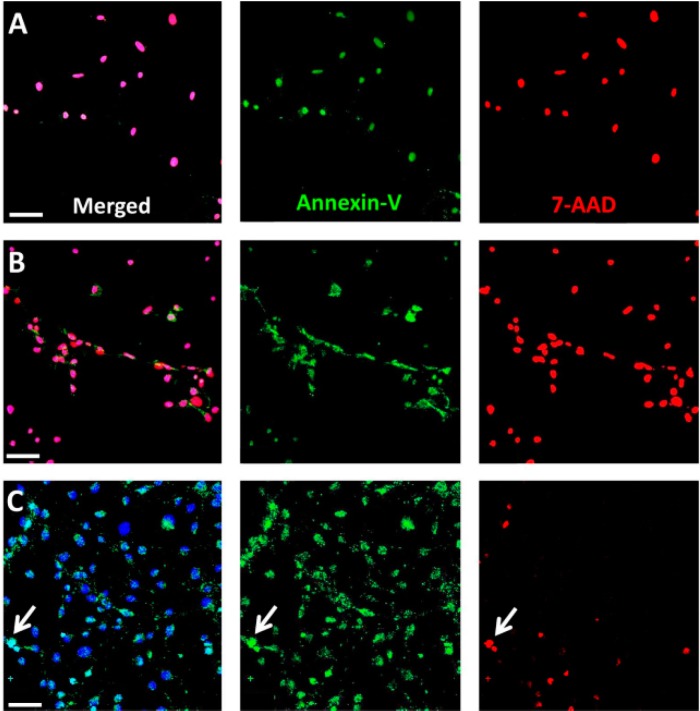
Staining for 7-AAD (red) and Annexin-V (green) was performed on NaIO_3_-treated RPE cells in order to distinguish between necrosis (both markers at any stage) and apoptosis (Annexin-V only at a certain stage). (**A**) NaIO_3_ treatment of RPE cells resulted in exclusively double-positive cells, indicating rapid necrotic cell death; (**B**) Incubation with ionomycin, which is used as a positive control for necrosis, showed similar results; (**C**) In contrast, treatment with staurosporine, an inducer of apoptosis, resulted in the majority of cells being only Annexin-V-positive cells. Only a few cells (arrow) were double positive for 7-AAD and Annexin-V (late apoptosis). Scale bar = 100 μm.

To further validate that the increase in cytotoxicity observed in the RPE cell culture in response to NaIO_3_ is related to necrosis, and not to conventional cell death, an analysis using 7-AAD and Annexin-V staining was performed. Thereby, necrotic cells are positive for both markers at any stage whereas apoptotic cells display Annexin-V only positivity at an early stage of cell death. Already 30 min post-exposure, NaIO_3_-treated (48 mM) RPE cells were doubly positive (Figure 6A), similar to necrotic cells after ionomycin treatment (0.1 mM; Figure 6B). On the other hand, staurosporine-treated (1 µM) RPE cells were only Annexin-V positive, with the exception of a few double-positive cells that represent a progressed apoptotic stage (Figure 6C).

**Figure 7:**
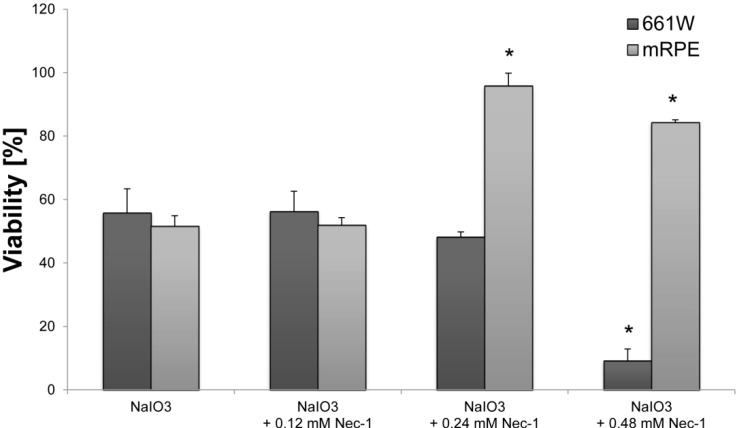
Necrostatin-1 is cytoprotective for NaIO_3_-treated RPE cells. Cell viability of 6 mM NaIO_3_-treated RPE cells was significantly increased after incubation with 0.24 (viability: 96%) and 0.48 mM Nec-1 (viability: 84%) compared to the control (viability: 51%). In contrast, the 661W cells were not rescued by the Nec-1 treatment. * *p* < 0.05.

In order to further clarify the involvement of necrosis in NaIO_3_-induced cell death, different concentrations of Necrostatin-1 (Nec-1), a compound that inhibits nonconventional cell death without interfering with caspase-dependent apoptosis [25,28], were administered to 6 mM NaIO_3_-treated RPE and 661W cultures concomitant to the NaIO_3_ treatment, and the cell viability was assessed for each concentration after 24 h (Figure 7). NaIO_3_ decreased the viability to 55.8% ± 7.6% in 661W cells and 50.5% ± 3.3% in RPE compared to the untreated controls. Treatment with 0.12 mM Nec-1 did not alter the outcome (56.2% ± 6.5% and 51.9% ± 2.4%, respectively), whereas 0.24 mM Nec-1 increased the viability of RPE cells significantly (95.9% ± 4.0%; *p* < 0.05). In contrast, NaIO_3_-treated 661W cells could not be rescued by treatment with Nec-1 (0.24 mM: 48.2% ± 1.6%). Similar results were found after treatment with 0.48 mM Nec-1 with 9.2% ± 3.7% and 84.3% ± 0.8% viability, respectively.

## 3. Discussion

The NaIO_3_ animal model of retinal degeneration is widely used in vision research to investigate retinal cell death. Treatment with NaIO_3_ results in reduced vision and is followed by RPE damage with concomitant photoreceptor degeneration [17]. However, the death mechanisms that are involved are still not fully elucidated. To identify additional details, we assessed the PRC death pathways *in vivo* and conducted an *in vitro* assessment of the effect of NaIO_3_ on both RPE cells and PRCs. Here, we report that NaIO_3_ *in vivo* induced rapid and pronounced degeneration of the RPE as well as a moderate and slow degeneration of the PRCs with a low number of TUNEL-positive cells in the ONL, starting at three days PI. We did not found TUNEL positivity in other retinal layers, for example the ganglion cell or the inner nuclear layer; nevertheless, we cannot exclude an effect of NaIO_3_ on those layers. The damaged PRCs *in vivo* were positive for cleaved caspase-3 as well as for activated calpain. Calpain is a calcium-dependent, non-lysosomal cysteine protease and was previously reported to be activated in 661W cells upon calcium overload in response to a nitric oxide donor [29]. In some photoreceptors of NaIO_3_-injected mice, calpain-positive cells co-expressed caspase-3, indicating that both cell-death modes occur concomitantly. That might represent the default pathway of PRC death, as similar results have also been observed in other retinal degeneration models *in vitro* [29] and *in vivo* [24]. A different explanation could be that calpain mediates caspase-dependent cell death in the PRCs by inducing cleavage and, thereby, activation of caspases.

*In vitro*, cell-death pathways were assessed in detail in RPE and 661W cells after NaIO_3_ treatment in order to differentiate between primary necrosis and conventional caspase-dependent apoptosis. An unambiguous identification of necrotic cell death is challenging, because of the absence of biochemical markers both *in vitro* and *in vivo*. However, the rupture of the plasma membrane with absence of caspase-3 positivity is a main indicator for ongoing necrotic cell death. This approach was pursued using the ApoToxGlo assay, which, besides viability and caspase-dependent cell death, measures the so-called dead-cell protease activity upon necrosis-induced loss of membrane integrity. Herein, we could provide evidence that NaIO_3_ treatment triggers apoptotic caspase-dependent cell death in the 661W cone-derived photoreceptor cell line, whereas necrotic cell death was induced in NaIO_3_-treated RPE cells. The observed direct effect on PRCs is supported by a recently published study using a NaIO_3_ concentration in the range of 250–750 µg/mL [16]. In our experiments, the lowest concentration used (6 mM or 1182 µg/mL) also resulted in 661W cell death. In contrast, we did not see a significant difference in vulnerability to NaIO_3_ when comparing primary RPE and 661W cells, only with respect to the mode of cell death. The fact that a high level of apoptosis is triggered in 661W cells, whereas only a few PRCs were apoptotic *in vivo*, might be related to the route of delivery. Although the *in vitro* administration resulted in direct exposure of the cells to the toxin, *in vivo*-administered NaIO_3_ reaches the PRCs through the blood–retina barrier (BRB) and the RPE, as the most adjacent cell type. The *Zonulae occludentes*, which are the anatomical basis of the BRB function of the RPE, were reported to be severely impaired by NaIO_3_ treatment [30].

Interestingly, primary PRCs seem to be less susceptible to the toxin than 661W cells, as their detected loss of viability upon NaIO_3_ treatment reached only approximately 50%, compared to 12.5% in the latter. This also supports a direct effect of NaIO_3_ on (rod) PRCs, but we were not able to distinguish the executed cell-death modes. However, as PRCs die *in vitro* if they lose contact to other retinal cells, our data using the primary PRC culture must be interpreted carefully, as ongoing cell death was already seen in untreated control cells. Differences in terms of the loss of cell viability between rod and cone PRCs have also been described in a recent study, suggesting that NaIO_3_ triggered the rapid and irreversible destruction of cones, whereas a decrease in the rod-evoked ERG response was subsequently rescued [4].

In conclusion, we herewith identified necrosis as the main pathway involved in NaIO_3_-induced RPE cell death. These results support earlier studies, observing morphological changes in the retina by OCT and electron microscopy [31,32]. The used intermediate concentration of 12 mM NaIO_3_ (2376 μg/mL) is comparable to the recently published data by Wang *et al.* [16], who demonstrated a cytotoxic effect of NaIO_3_ on ARPE19 cells, an immortalized human RPE cell line that displays RPE features *in vitro* [33]. As it is possible that apoptotic cells undergo necrosis in a secondary stage *in vitro*, owing to the lack of macrophages, we performed an analysis on NaIO_3_-treated RPE cells at early time points post-exposure, confirming necrotic cell death by the concomitant presence of Annexin-V and 7-AAD. Consistently, NaIO_3_-induced RPE degeneration *in vitro* was significantly reduced by Nec-1, an inhibitor of the nonconventional, necrotic-like cell death under caspase-compromised conditions [34]. Nec-1 has been shown to decrease the production of reactive oxygen species (ROS) [35], levels of which have been reported to be elevated in NaIO_3_-treated RPE cells [18]. Protection against ROS formation could, therefore, contribute to the significant rescue effect of Nec-1 observed in NaIO_3_-treated RPE cells. However, further investigations will be necessary in order to shed light on the exact mechanisms by which Nec-1 is able to rescue NaIO_3_-treated RPE cells. Alternative effects of NaIO_3_ on the RPE, in addition to the induction of oxidative stress [18], could involve mitochondrial dysfunction and upregulation of p62 [36].

## 4. Experimental Section

### 4.1. Animal Treatment

C57BL/6J mice (4–6 weeks old, Charles River, Germany) were treated according to the ARVO Statement for the Use of Animals in Ophthalmic and Vision Research, following approval from the Commission for Animal Experimentation of the Canton of Bern, Switzerland. A single intravenous injection of sterile 1% NaIO_3_ (35 mg/kg bodyweight) in 0.9% NaCl was performed. Control animals received a single intravenous injection of 0.9% NaCl. Animals were sacrificed at different time points PI, and the eyes were then enucleated for electron microscopy (EM), immunohistochemistry (IHC), and ELISA-based protein activity assays.

### 4.2. Cell Culture

To assess the effect of NaIO_3_ on retinal cells *in vitro*, the murine photoreceptor cell line 661W (kindly provided by M. Al-Ubaidi, University of Oklahoma, Oklahoma City, OK, USA) was used [37]. The cells were propagated under normal culture conditions in Dulbecco’s modified Eagle’s medium (DMEM) supplemented with GlutaMAX and 1% antibiotic/antimycotic + 10% fetal bovine serum (FBS; Life Technologies, Carlsbad, CA, USA).

Primary mouse RPE cells were enzymatically isolated from 10-day-old wild-type (WT) mice. Briefly, enucleated eyes were incubated with 2% dispase (Life Technologies) in phosphate-buffered salt solution (PBS; Life Technologies) with gentle shaking at 37 °C for 25 min. The anterior half of the eye and the vitreous part were discarded. The retina was gently floated off of the RPE with PBS and removed by cutting the optic nerve. The RPE cells were mechanically harvested, centrifuged, and re-suspended in DMEM + 10% FBS. The epithelial origin of the prepared cells was determined by staining for the tight-junction protein ZO-1 and F-actin (see Figure S2). RPE cells were used at low passage up to passage 6.

The primary culture of photoreceptors was prepared from retinal tissue of postnatal day 4–8 WT animals using the Papain Dissociation System (Worthington Biochemical, Lakewood, NJ, USA), according to the manufacturer’s instruction. After the last centrifugation step, the cells were re-suspended in neurobasal medium supplemented with 2% B27, 1% N2, 1% antibiotic/antimycotic as well as 0.8 mM Glutamine (Life Technologies).

### 4.3. Electron Microscopy

Eyes were enucleated and fixed with Karnowsky fixation medium (1% paraformaldehyde (PFA); Sigma-Aldrich, Buchs, Switzerland), 3% sodium cacodylate–HCl (Science Services, Munich, Germany), and 3% glutaraldehyde (Sigma-Aldrich) for 24 h. Following the removal of the lens, post-fixation, and washing in EM buffer (2.5% glutaraldehyde (Sigma-Aldrich) and 0.1 M sodium cacodylate–HCl (Science Services)) for 15 min, the eyes were post-fixed in 4% osmium tetroxide (Science Services) for 15 min. The tissue was dehydrated, washed with a resin/1,2-propylene oxide mixture (Merck, Darmstadt, Germany) three times for 20 min each, and infiltrated with resin overnight. The resin-embedded tissue was then trimmed and cut into 80-nm thick sections (Ultracut, Reichert Microscope Services, Depew, NY, USA) using an Ultra-45 °C diamond knife (Diatome, Biel, Switzerland). Sections were applied to copper grids (G100H-C3; Science Services) and contrasted with 0.1% lead citrate (Science Services). EM analyses were performed on a CM 12 electron microscope (Philips Applied Technologies, Eindhoven, The Netherlands).

### 4.4. Immunohistochemistry

Caspase detection: Fixed and dehydrated eyes of treated and untreated mice were embedded in paraffin. Tissue sections with a thickness of five µm were cut. Antigen retrieval was performed using sodium citrate buffer (10 mM sodium citrate, 0.05% Tween-20, pH 6.0; Sigma-Aldrich) in a pressure cooker for five min. Sections were then permeabilized using 0.2% Triton X-100 in Tris-buffered saline (TBS; Sigma-Aldrich) for 20 min, and blocked 1% bovine serum albumin (BSA; Sigma-Aldrich) and 10% normal goat serum (NGS; DAKO, Baar, Switzerland) for 1 h before an overnight incubation with the primary antibody (rabbit anti-cleaved-caspase-3; 1:100; Cell signaling, Leiden, The Netherlands) at 4 °C. After washing, incubation in a secondary antibody (goat anti-rabbit Alexa Fluor^®^ 594 or 488 (Life Technologies), 1:500) was performed for 45 min. DAPI (Vector Laboratories, Burlingame, CA, USA) or Sytox Green (Life Technologies) was used to counterstain the cell nuclei.

Calpain activity: Unfixed eyes were snap-frozen in Tissue-Tek O.C.T Compound (Sakura, Alphen aan den Rijn, The Netherlands) and 7-μm cryosections were processed to visualize calpain activity, according to Paquet-Durand, *et al*., 2006 [23]. All cell outlines are labelled, with calpain activity-positive cells displaying a bright labelling in the nucleus and cytosol. A fluorescent calpain substrate 7-amino-4-chloromethylcoumarin, *t-*BOC-l-leucyl-l-methionine amide (CMAC, *t*-BOC-Leu-Met; Life Technologies), was added at a final concentration of 2 µm, and then incubated in the dark for 1 h at 35 °C. Sytox Green (Life Technologies) was used as a nuclear counterstain. Calpain-positive cells were counted manually in the visual field by the cell-counter plug-in for ImageJ, which was calculated as a percentage of positive cells with respect to all photoreceptor cells in the ONL. To assess TUNEL and calpain or caspase-3 and calpain activity in co-staining, sections stained for calpain activity were subsequently fixed in freshly prepared 4% PFA at RT for 15 min before applying the TUNEL kit or performing antibody staining, respectively.

RPE-specific markers: Mouse primary RPE cells (50,000 cells/well) grown on fibronectin-coated (10 µg/mL) eight-well chamber slides (Lab-Tek™ II, Sigma-Aldrich) were fixed with 4% PFA for 15 min, washed in PBS, and blocked with 1% BSA in PBS supplemented with 10% NGS for 1 h. Immunohistochemistry using rabbit anti-ZO-1 and Texas Red conjugated phalloidin was performed as described above.

TUNEL assay: Staining was performed using the *in situ* cell-death detection kit, TMRed (Roche Applied Sciences, Basel, Switzerland) on either cryo- or paraffin sections. Staining was carried out according to the manufacturer’s instructions. Confocal microscopy was performed, either by the Zeiss scanning laser microscope (Zeiss LSM710; Carl Zeiss Microscopy, Jena, Germany) or a Leica SP2 (Leica Microsystems, Heerbrugg, Switzerland).

### 4.5. Calpain and Caspase Activity Assays

The calpain and caspase activity was assessed on protein lysates of treated and untreated retinas using enzyme activity assay kits (BioVision, Milpitas, CA, USA) according to the manufacturer’s instructions. Animals were sacrificed at days 3, 7, and 10 PI of 35 mg/kg NaIO_3_, and the retinas were harvested and pooled (*n* ≥ 4). Following homogenization of the retina and subsequent cell lysis in RIPA buffer (150 mM NaCl, 1.0% IGEPAL^®^, 0.5% sodium deoxycholate, 0.1% SDS, 50 mM Tris, pH 8.0; Sigma-Aldrich) supplemented with inhibitors to prevent protein degradation through non-caspase family proteases (Complete Mini; Roche Applied Science) on ice for 30 min, the lysates were centrifuged at 13,000 rpm at 4 °C for 20 min. In the supernatant, the protein content was assessed using the DC protein assay (BioRad, Cressier, Switzerland), and 50 µg of retinal lysate proteins (triplicates) were incubated in reaction buffer in a well of a 96-well plate. The samples were incubated for 1 h with the specific substrate, which emits a yellow–green fluorescence upon cleavage in the presence of the corresponding caspase/calpain. The cleaved substrate was then fluorometrically measured at 505 nm using a multimode plate reader (Infinite 200PRO; Tecan, Männedorf, Switzerland). Activity data from NaIO_3_-treated animals were plotted against control values obtained from NaCl-injected control mice for each specific time point. Results are expressed as fold increase in enzyme activity. In order to confirm a specific activity after NaIO_3_ treatment, three independent retinal lysate samples of NaIO_3_-treated mice were supplemented with 100 µM of the inhibitor Z-LLY-FMK (BioVision, Milpitas, CA, USA) for 1 h before incubation with the substrate.

### 4.6. ApoTox Glo Triplex Assay

The ApoTox Glo Triplex assay (Promega, Madison, WI, USA) was used to simultaneously assess viability, cytotoxicity, and caspase-dependent apoptosis in response to various concentrations of NaIO_3_ *in vitro.* A 1 µM staurosporine solution (Sigma-Aldrich) was used as a positive control for apoptosis, and cells were sonicated prior to plating in order to rupture the cell membrane as positive control for necrosis. For the experimental setup, 661W photoreceptors and primary RPE cells were seeded at a density of 10,000 cells/well (30,000 cells/cm^2^). The next day, the cells were sonicated or treated with either staurosporine (1 μM), different concentrations of NaIO_3_ (6, 12, or 48 mM), or left untreated to act as controls. At different time points post-treatment (6, 14, and 24 h), the ApoTox Glo assay was performed according to the manufacturer’s instructions. Cell viability (wavelength sets: 400_Ex_/505_Em_) and cytotoxicity (wavelength sets: 485_Ex_/520_Em_) were measured using a microplate reader (Tecan Infinite 200Pro). At each time point, a second measurement followed, using the Caspase-Glo^®^ Assay Technology (Promega), for caspase-dependent cell death by adding a luminogenic caspase-3/-7 substrate to the cell culture medium. Following incubation at room temperature for 1 h, the luminescence was measured with an integration time setting of between 0.5 and 1 s, using the Tecan Infinite 200Pro reader.

### 4.7. Annexin-V and 7-AAD Staining

To confirm primary necrosis in RPE cells *in vitro*, we used a staining kit for 7-AAD and Annexin-V. Necrotic cells are positive for both markers at any stage, whereas early apoptotic cells include a stage at which cells are exclusively Annexin-V positive. RPE and 661W cells were seeded on fibronectin-coated (10 µg/mL) chamber slides (Lab-Tek™ II, Sigma-Aldrich; 20,000 cells/well) for 24 h before treatment with NaIO_3_ (48 mM). Staurosporine (1 µM) and ionomycin (0.1 mM; Sigma-Aldrich) treatment was employed as positive controls. Cells were washed and incubated in the 7-AAD/Annexin-V reaction mix and incubated for 15 min before fixation with 2% PFA. Counterstaining was performed using the Hoechst stain NucBlue^®^ Live Ready Probes Reagent^®^ (Life Technologies).

### 4.8. Necrostatin-1 Treatment

Nec-1 (Enzo Life Sciences (Farmingdale, NY, USA)) was dissolved in DMSO according to the manufacturer and further diluted in DMEM to achieve the desired concentrations (0.12, 0.24, 0.48 mM). DMSO was also added to control cells (at the dissolvent concentration used) in order to exclude a cytotoxic effect of the solvent. Nec-1 was administered concomitantly with the NaIO_3_ and the XTT viability assay (Roche Life Science) was performed 24 h later according to the manufacturer’s instructions. Blank values with Nec-1 but without cells were subtracted in order to correct for its color-related absorbance.

### 4.9. Statistical Analysis

Three individual experiments were performed (mean ± SD) and a statistical evaluation using analysis of variance (ANOVA) was executed in SPSS software 20 (IBM, Hampshire, UK), followed by a multiple-comparisons *post hoc* test to determine the significant differences between the mean values. Differences were considered significant at *p* ≤ 0.05.

## 5. Conclusions

In conclusion, the NaIO_3_ mouse model of retinal degeneration provides a highly complex system of different and concomitantly acting cell-death pathways, affecting both RPE and neurosensory retina. Owing to this complexity, it mimics the multilayered situation within a degenerating retina and could, therefore, serve as the model of choice for the investigation of the efficiency of combined therapeutic approaches. We hereby suggest that interference with the NaIO_3_-induced cell death in the mouse eye *in vivo* can only be achieved using a combinatory treatment consisting of inhibitors of both conventional as well as unconventional cell death. Specifically, we believe that the administration of RPE and/or PRC precursor cells as a cell therapy, supported by the administration of neuroprotective substances preventing both conventional and nonconventional cell death, could represent the most straightforward approach to reverse or prevent the loss of vision. This research might help to design future therapeutic approaches towards successful AMD therapy.

## Supplementary Materials

Supplementary materials can be found at http://www.mdpi.com/1422-0067/16/07/15086/s1.

## References

[1] Webster S.H., Rice M.E., Highman B., von Oettingen W.F. (1957). The toxicology of potassium and sodium iodates: Acute toxicity in mice. J. Pharmacol. Exp. Ther..

[2] Mizota A., Adachi-Usami E. (1997). Functional recovery of retina after sodium iodate injection in mice. Vis. Res..

[3] Higuchi M., Tomioka M., Takano J., Shirotani K., Iwata N., Masumoto H., Maki M., Itohara S., Saido T.C. (2005). Distinct mechanistic roles of calpain and caspase activation in neurodegeneration as revealed in mice overexpressing their specific inhibitors. J. Biol. Chem..

[4] Machalinska A., Kawa M.P., Pius-Sadowska E., Roginska D., Klos P., Baumert B., Wiszniewska B., Machalinski B. (2013). Endogenous regeneration of damaged retinal pigment epithelium following low dose sodium iodate administration: An insight into the role of glial cells in retinal repair. Exp. Eye Res..

[5] Gong L., Wu Q., Song B., Lu B., Zhang Y. (2008). Differentiation of rat mesenchymal stem cells transplanted into the subretinal space of sodium iodate-injected rats. Clin. Exp. Ophthalmol..

[6] Korte G.E., Reppucci V., Henkind P. (1984). RPE destruction causes choriocapillary atrophy. Investig. Ophthalmol. Vis. Sci..

[7] Strauss O. (2005). The retinal pigment epithelium in visual function. Physiol. Rev..

[8] Bazan N.G. (2008). Neurotrophins induce neuroprotective signaling in the retinal pigment epithelial cell by activating the synthesis of the anti-inflammatory and anti-apoptotic neuroprotectin D1. Adv. Exp. Med. Biol..

[9] Franco L.M., Zulliger R., Wolf-Schnurrbusch U.E., Katagiri Y., Kaplan H.J., Wolf S., Enzmann V. (2009). Decreased visual function after patchy loss of retinal pigment epithelium induced by low-dose sodium iodate. Investig. Ophthalmol. Vis. Sci..

[10] Sen H.A., Berkowitz B.A., Ando N., de Juan E. (1992). *In vivo* imaging of breakdown of the inner and outer blood-retinal barriers. Investig. Ophthalmol. Vis. Sci..

[11] Baich A., Ziegler M. (1992). The effect of sodium iodate and melanin on the formation of glyoxylate. Pigment Cell Res..

[12] Stern W.H., Ernest J.T., Steinberg R.H., Miller S.S. (1980). Interrelationships between the retinal pigment epithelium and the neurosensory retina. Aust. J. Ophthalmol..

[13] Ashburn F.S., Pilkerton A.R., Rao N.A., Marak G.E. (1980). The effects of iodate and iodoacetate on the retinal adhesion. Investig. Ophthalmol. Vis. Sci..

[14] Yoon Y.H., Marmor M.F. (1993). Retinal pigment epithelium adhesion to bruch’s membrane is weakened by hemicholinium-3 and sodium iodate. Ophthalmic Res..

[15] Tao Z., Dai J., He J., Li C., Li Y., Yin Z.Q. (2013). The influence of NaIO_3_-induced retinal degeneration on intra-retinal layer and the changes of expression profile/morphology of DA-ACs and mRGCs. Mol. Neurobiol..

[16] Wang J., Iacovelli J., Spencer C., Saint-Geniez M. (2014). Direct effect of sodium iodate on neurosensory retina. Investig. Ophthalmol. Vis. Sci..

[17] Qin S., Lu Y., Rodrigues G.A. (2014). Resveratrol protects rpe cells from sodium iodate by modulating pparalpha and ppardelta. Exp. Eye Res..

[18] Zhou P., Ye H.F., Jiang Y.X., Yang J., Zhu X.J., Sun X.H., Luo Y., Dou G.R., Wang Y.S., Lu Y. (2012). αA crystallin may protect against geographic atrophy-meta-analysis of cataract *vs.* cataract surgery for geographic atrophy and experimental studies. PLoS ONE.

[19] Debnath J., Baehrecke E.H., Kroemer G. (2005). Does autophagy contribute to cell death?. Autophagy.

[20] Sperandio S., de Belle I., Bredesen D.E. (2000). An alternative, nonapoptotic form of programmed cell death. Proc. Natl. Acad. Sci. USA.

[21] Syntichaki P., Xu K., Driscoll M., Tavernarakis N. (2002). Specific aspartyl and calpain proteases are required for neurodegeneration in *C. elegans*. Nature.

[22] Yamashima T., Kohda Y., Tsuchiya K., Ueno T., Yamashita J., Yoshioka T., Kominami E. (1998). Inhibition of ischaemic hippocampal neuronal death in primates with cathepsin B inhibitor CA-074: A novel strategy for neuroprotection based on “calpain-cathepsin hypothesis”. Eur. J. Neurosci..

[23] Paquet-Durand F., Azadi S., Hauck S.M., Ueffing M., van Veen T., Ekstrom P. (2006). Calpain is activated in degenerating photoreceptors in the rd1 mouse. J. Neurochem..

[24] Kaur J., Mencl S., Sahaboglu A., Farinelli P., van Veen T., Zrenner E., Ekstrom P., Paquet-Durand F., Arango-Gonzalez B. (2011). Calpain and PARP activation during photoreceptor cell death in P23H and S334ter rhodopsin mutant rats. PLoS ONE.

[25] Trichonas G., Murakami Y., Thanos A., Morizane Y., Kayama M., Debouck C.M., Hisatomi T., Miller J.W., Vavvas D.G. (2010). Receptor interacting protein kinases mediate retinal detachment-induced photoreceptor necrosis and compensate for inhibition of apoptosis. Proc. Natl. Acad. Sci. USA.

[26] Carido M., Zhu Y., Postel K., Benkner B., Cimalla P., Karl M.O., Kurth T., Paquet-Durand F., Koch E., Münch T. (2014). Characterization of a mouse model with complete RPE loss and its use for RPE cell transplantation. Investig. Ophthalmol. Vis. Sci..

[27] Nakagawa T., Zhu H., Morishima N., Li E., Xu J., Yankner B.A., Yuan J. (2000). Caspase-12 mediates endoplasmic-reticulum-specific apoptosis and cytotoxicity by amyloid-β. Nature.

[28] Mead B., Berry M., Logan A., Scott R.A., Leadbeater W., Scheven B.A. (2015). Stem cell treatment of degenerative eye disease. Stem Cell Res..

[29] Sanvicens N., Gomez-Vicente V., Masip I., Messeguer A., Cotter T.G. (2004). Oxidative stress-induced apoptosis in retinal photoreceptor cells is mediated by calpains and caspases and blocked by the oxygen radical scavenger CR-6. J. Biol. Chem..

[30] Ringvold A., Olsen E.G., Flage T. (1981). Transient breakdown of the retinal pigment epithelium diffusion barrier after sodium iodate: A fluorescin angiographic study in the rabbit. Exp. Eye Res..

[31] Hariri S., Tam M.C., Lee D., Hileeto D., Moayed A.A., Bizheva K. (2013). Noninvasive imaging of the early effect of sodium iodate toxicity in a rat model of outer retina degeneration with spectral domain optical coherence tomography. J. Biomed. Opt..

[32] Kiuchi K., Yoshizawa K., Shikata N., Moriguchi K., Tsubura A. (2002). Morphologic characteristics of retinal degeneration induced by sodium iodate in mice. Curr. Eye Res..

[33] Ablonczy Z., Dahrouj M., Tang P.H., Liu Y., Sambamurti K., Marmorstein A.D., Crosson C.E. (2011). Human retinal pigment epithelium cells as functional models for the RPE *in vivo*. Investig. Ophthalmol. Vis. Sci..

[34] Degterev A., Huang Z., Boyce M., Li Y., Jagtap P., Mizushima N., Cuny G.D., Mitchison T.J., Moskowitz M.A., Yuan J. (2005). Chemical inhibitor of nonapoptotic cell death with therapeutic potential for ischemic brain injury. Nat. Chem. Biol..

[35] Xu X., Chua C.C., Kong J., Kostrzewa R.M., Kumaraguru U., Hamdy R.C., Chua B.H. (2007). Necrostatin-1 protects against glutamate-induced glutathione depletion and caspase-independent cell death in HT-22 cells. J. Neurochem..

[36] Juel H.B., Faber C., Svendsen S.G., Vallejo A.N., Nissen M.H. (2013). Inflammatory cytokines protect retinal pigment epithelial cells from oxidative stress-induced death. PLoS ONE.

[37] Tan E., Ding X.P., Saadi A., Agarwal N., Naash M.I., Al-Ubaidi M.R. (2004). Expression of cone-photoreceptor-specific antigens in a cell line derived from retinal tumors in transgenic mice. Investig. Ophthalmol. Vis. Sci..

