# Supplementary material for: Retinal Cell Death Caused by Sodium Iodate Involves Multiple Caspase-Dependent and Caspase-Independent Cell-Death Pathways
Source: Int J Mol Sci. 2015 Jul 3;16(7):15086–103. doi: 10.3390/ijms160715086 (PMC4519888; doi:10.3390/ijms160715086)
Supplement: Supplementary file 1 [file ijms-16-15086-s001.pdf]

Supplementary Information

In order to investigate the effect of $NaIO_3$ on primary (rod) PRCs retinas from postnatal day 4 C57BL6 mice were dissociated using the papain dissociation kit according to the manufacturer's instruction (Worthington Biochemical Corporation, Lakewood, NJ, USA). Single cells were then seeded at 10,000 cells/well in a 96-well plate. One day later, the cells were incubated with different concentrations of $NaIO_3$ for 24 h.

In the dissociated retinal cell culture that consists of roughly 35%–50% PRCs the cells adapted a more round morphology with increasing concentrations of $NaIO_3$ (6 or 48 mM) administered. The apoptosis inducer staurosporine triggered similar outcome (Figure S1A–D).

To quantify the loss of cell viability in primary photoreceptors the MTT (3-(4,5-dimethylthiazol-2-yl)-2,5-diphenyltetrazolium bromide) viability assay was employed. A maximum of 50% loss in cell viability was observed with 48 mM $NaIO_3$ 14 h after exposure with the toxin (Figure S1E). Because the dissociated primary cells are dying rapidly in culture as a result of the absence of physiological environment we have performed the PCD experiments with the 661W cell line only.

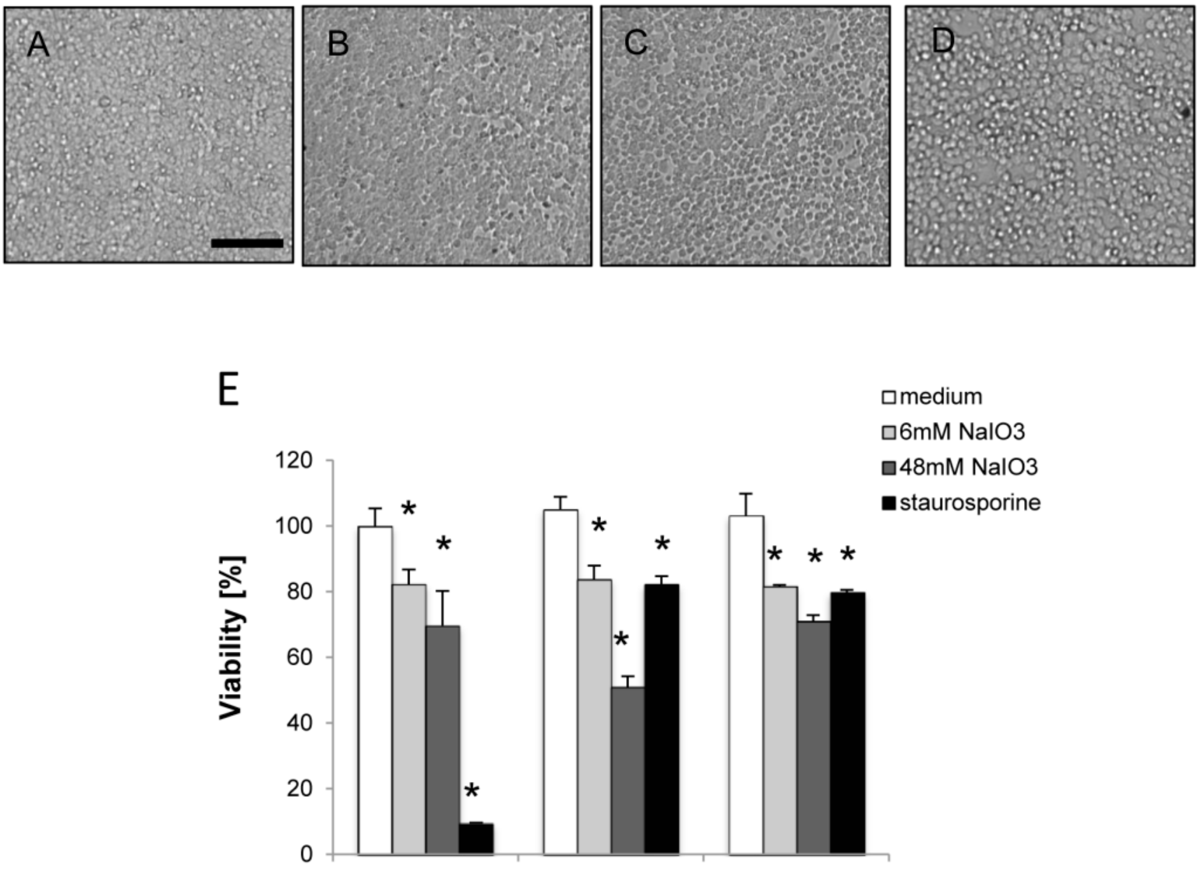

Figure S1. Dissociated retinal cells from the neurosensory retina containing approx. 50% photoreceptors were exposed to $NaIO_3$. Untreated controls (**A**) and treated cells (6 mM (**B**), 48 mM (**C**) or 1 µM staurosporine (**D**)) imaged at 14 h post treatment. Degenerating cells are characterized by roundish cell morphology. Scale = 50 µm; and (**E**) Viability assay indicates a significant loss of viability for all treatments (* $p \leq 0.05$).

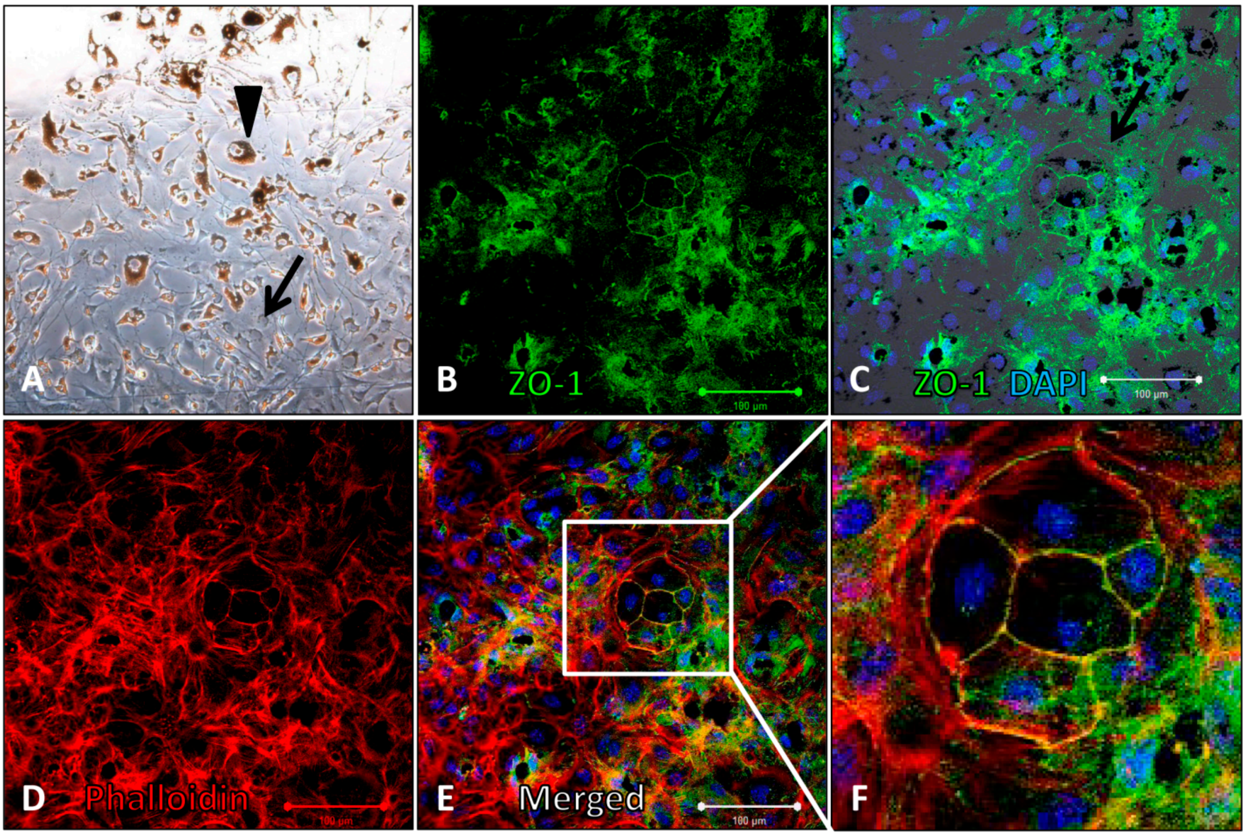

Figure S2. Characterization of murine RPE cells. (**A**) Freshly isolated RPE cells display melanin pigmentation (arrow) and hexagonal morphology. They are surrounded by fibroblast-like RPE cells (arrowhead) that underwent epithelial mesenchymal transition; (**B**,**C**) RPE cells with cobblestone-like morphology (arrow) expressed the tight junction marker ZO-1 (green); (**D**) The epithelial nature of the cells was confirmed by F-actin staining using phalloidin (red); (**E**) Merged image to visualize the co-localization of all investigated RPE markers with a magnification of the central area in panel (**F**).
